# Supplementary material for: Effective descriptions of bosonic systems can be considered complete
Source: Nat Commun. 2025 Nov 6;16:9744. doi: 10.1038/s41467-025-64872-3 (PMC12592358; doi:10.1038/s41467-025-64872-3)
Supplement: Supplementary file 1 — Supplementary Information [file 41467_2025_64872_MOESM1_ESM.pdf]

Supplementary Information - Effective descriptions of bosonic systems can be considered complete

Francesco Arzani,[1] Robert I. Booth,[2,3] and Ulysse Chabaud[1]
[1]DIENS, École Normale Supérieure, PSL University, CNRS, INRIA, France
[2]University of Edinburgh, United Kingdom
[3]University of Bristol, United Kingdom

I. PROOF OF THEOREM 1: EFFECTIVE DIMENSION OF PHYSICAL UNITARY CHANNELS

Hereafter, we denote by $\Pi_N = \sum_{n=0}^N |n\rangle\langle n|$ the projector onto the subspace of states with a number of particle at most N, for $N \geq 0$. We give a formal version of Theorem 1 from the main text:

Theorem 1 (Effective dimension of physical unitary channels). *Let $\mathcal{U} = \hat{U} \cdot \hat{U}^\dagger$ be a unitary channel, with $\hat{U} \in \mathcal{U}(\mathcal{S})$ a physical unitary operator. Let $E \geq 0$ be an energy bound, and let $\epsilon > 0$ be an approximation parameter. Then, there exists $N = \mathcal{O}\big(\frac{E}{\epsilon^4} E_{\hat{U}}\big(\frac{64E}{\epsilon^2}\big)\big) \in \mathbb{N}$ and a unitary operator $\hat{V}_N$ over the range of Π_N that approximates $\hat{U}$ in the following sense: for any unitary operator $\hat{V}'$ on $\mathrm{Range}(I - \Pi_N)$, denoting $\hat{V} = \hat{V}_N \oplus \hat{V}'$ and $\mathcal{V} = \hat{V} \cdot \hat{V}^\dagger$,*

$$\|\mathcal{U} - \mathcal{V}\|_\diamond^E \leq \epsilon, \tag{1}$$

where the energy-constrained diamond norm is defined with respect to the number Hamiltonian $\hat{n} = \hat{a}^\dagger \hat{a}$. Moreover, the cut-off operator $\hat{V}_N$ can be computed from $\hat{U}$ in time

$$\mathcal{O}\left(\frac{NE^2}{\epsilon^4}\right) = \mathcal{O}\left(\frac{E^3}{\epsilon^8} E_{\hat{U}}\left(\frac{64E}{\epsilon^2}\right)\right). \tag{2}$$

Proof sketch. We focus on the single-mode case for simplicity. With the notation of the theorem, by [1, Theorem 1], it is equivalent to show that there exists $\hat{V}_N$ such that for all $\hat{V}'$,

$$\sup_{\substack{|\psi\rangle \in \mathcal{H} \\ \langle\psi|\hat{n}|\psi\rangle \leq E}} \|\hat{U}|\psi\rangle\langle\psi|\hat{U}^\dagger - (\hat{V}_N \oplus \hat{V}')|\psi\rangle\langle\psi|(\hat{V}_N^\dagger \oplus \hat{V}'^\dagger)\|_1 \leq \epsilon. \tag{3}$$

As explained in the Methods section of the main text, the idea of the proof is to perform two successive cut-offs of the state space, before and after the unitary evolution, respectively parametrised by integers M and N, and to carefully control the approximation at each step, followed by a rounding of the cut-off operator to the closest unitary operator. More precisely, the steps of the proof are as follows: let $N \geq M \geq 0$,

(i) show that the supremum over energy-constrained states is well approximated by a supremum over $\mathcal{H}_M$ when M is large, using the gentle measurement lemma;

(ii) show that the action of $\hat{U}$ on $\mathcal{H}_M$ is well-approximated by that of $\Pi_N \hat{U} \Pi_N$ when N is large, also using the gentle measurement lemma;

(iii) show that the action of $\Pi_N \hat{U} \Pi_N$ itself on $\mathcal{H}_M$ is well-approximated by that of a unitary operator $\hat{V}_N$ defined on $\mathcal{H}_N \supset \mathcal{H}_M$ when N is large, using the Gram–Schmidt orthonormalisation procedure;

(iv) combine the successive approximations to conclude.

Proof of Theorem 1. (i) Let $\hat{V}$ be a unitary operator and let $|\psi\rangle = \sum_{n\geq 0} \psi_n |n\rangle \in \mathcal{H}$ such that $\langle\psi|\hat{n}|\psi\rangle \leq E$. Denoting $\mathcal{U} = \hat{U} \cdot \hat{U}^\dagger$ and $\mathcal{V} = \hat{V} \cdot \hat{V}^\dagger$, we have

$$\begin{aligned}
\|(\mathcal{U} - \mathcal{V})(\psi)\|_1 &\leq \|\hat{U}\Pi_M|\psi\rangle\langle\psi|\Pi_M\hat{U}^\dagger - \hat{V}\Pi_M|\psi\rangle\langle\psi|\Pi_M\hat{V}^\dagger\|_1 \\
&\quad + \|\hat{U}(|\psi\rangle\langle\psi| - \Pi_M|\psi\rangle\langle\psi|\Pi_M)\hat{U}^\dagger - \hat{V}(|\psi\rangle\langle\psi| - \Pi_M|\psi\rangle\langle\psi|\Pi_M)\hat{V}^\dagger\|_1 \\
&\leq \|\hat{U}\Pi_M|\psi\rangle\langle\psi|\Pi_M\hat{U}^\dagger - \hat{V}\Pi_M|\psi\rangle\langle\psi|\Pi_M\hat{V}^\dagger\|_1 \\
&\quad + \|\hat{U}(|\psi\rangle\langle\psi| - \Pi_M|\psi\rangle\langle\psi|\Pi_M)\hat{U}^\dagger\|_1 \\
&\quad + \|\hat{V}(|\psi\rangle\langle\psi| - \Pi_M|\psi\rangle\langle\psi|\Pi_M)\hat{V}^\dagger\|_1 \\
&= \|(\mathcal{U} - \mathcal{V})(\Pi_M\psi\Pi_M)\|_1 + 2\||\psi\rangle\langle\psi| - \Pi_M|\psi\rangle\langle\psi|\Pi_M\|_1 \\
&\leq \|(\mathcal{U} - \mathcal{V})(\Pi_M\psi\Pi_M)\|_1 + 4\sqrt{\frac{E}{M}},
\end{aligned} \tag{4}$$

where we used the triangle inequality for the first two steps, the fact that the trace distance is unitarily invariant in the third step, and the gentle measurement lemma in the last step.

(ii) Given $\hat{U} \in \mathcal{U}(\mathcal{S})$ and $M \geq 0$, we now look for $\hat{V}$ such that $\|(\mathcal{U}-\mathcal{V})(\phi)\|_1$ is uniformly bounded over $\text{Range}(\Pi_\text{M})$. With $N \geq M$, we pick a unitary operator of the form $\hat{V} = \hat{V}_N \oplus \hat{V}'$, where $\hat{V}_N$ is a unitary operator over $\mathcal{H}_N$ to be defined later and $\hat{V}'$ is arbitrary. For all $|\phi\rangle \in \mathcal{H}_M$, we write $|\phi_U\rangle := \hat{U}|\phi\rangle$ and $|\phi_V\rangle := \hat{V}|\phi\rangle = \hat{V}_N|\phi\rangle$. We have $\Pi_N |\phi_V\rangle\langle\phi_V| \Pi_N = |\phi_V\rangle\langle\phi_V|$, since $\hat{V} = \hat{V}_N \oplus \hat{V}'$ and $N \geq M$, and $\langle\phi_U| \hat{n} |\phi_U\rangle \leq E_{\hat{U}}(M)$, where

$$E_{\hat{U}}(M) := \sup_{|\chi\rangle \in \mathcal{H}_M} \langle\chi| \hat{U}^\dagger \hat{n} \hat{U} |\chi\rangle , \tag{5}$$

which is a bounded quantity for each $M \geq 0$ since $\hat{U} \in \mathcal{U}(\mathcal{S})$ and $\mathcal{H}_M$ is compact. Hence, following the same steps as in Eq. (4), for all $N \geq M$,

$$\begin{aligned}
\|(\mathcal{U}-\mathcal{V})(\phi)\|_1 &\leq \|\Pi_N |\phi_U\rangle\langle\phi_U| \Pi_N - \Pi_N |\phi_V\rangle\langle\phi_V| \Pi_N\|_1 \\
&\quad + \| |\phi_U\rangle\langle\phi_U| - \Pi_N |\phi_U\rangle\langle\phi_U| \Pi_N\|_1 \\
&\quad + \| |\phi_V\rangle\langle\phi_V| - \Pi_N |\phi_V\rangle\langle\phi_V| \Pi_N\|_1 \\
&\leq \|\Pi_N |\phi_U\rangle\langle\phi_U| \Pi_N - \Pi_N |\phi_V\rangle\langle\phi_V| \Pi_N\|_1 + 2\sqrt{\frac{E_{\hat{U}}(M)}{N}} \\
&= \|\Pi_N \hat{U} |\phi\rangle\langle\phi| \hat{U}^\dagger \Pi_N - \hat{V}_N |\phi\rangle\langle\phi| \hat{V}_N^\dagger\|_1 + 2\sqrt{\frac{E_{\hat{U}}(M)}{N}}.
\end{aligned} \tag{6}$$

where we used the triangle inequality for the first step, and the fact that $\Pi_N |\phi_V\rangle\langle\phi_V| \Pi_N = |\phi_V\rangle\langle\phi_V|$ and the gentle measurement lemma for the second step.

(iii) Given $\hat{U} \in \mathcal{U}(\mathcal{S})$ and $N \geq M \geq 0$, we now look for a unitary operator $\hat{V}_N$ over $\mathcal{H}_N$ such that $\|\Pi_N \hat{U} |\phi\rangle\langle\phi| \hat{U}^\dagger \Pi_N - \hat{V}_N |\phi\rangle\langle\phi| \hat{V}_N^\dagger\|_1$ is uniformly bounded over $\mathcal{H}_M$. We first derive useful properties of the column vectors $(|u_0\rangle, \dots, |u_M\rangle)$ of $\Pi_N \hat{U} \Pi_M$ (i.e., the first M columns of $\Pi_N \hat{U} \Pi_N$), which are obtained in Eqs. (11,12) and Lemma 1.

Let us denote $u_{ij} := \langle i|\hat{U}|j\rangle$ the Fock basis coefficients of the unitary operator $\hat{U}$, for all $i, j \in \mathbb{N}$. For all $k = 0, \dots, M$, we have

$$\begin{aligned}
\langle u_k|u_k\rangle &= \sum_{n=0}^{N} |u_{nk}|^2 \\
&= 1 - \sum_{n>N} |u_{nk}|^2 \\
&\leq 1,
\end{aligned} \tag{7}$$

since $\hat{U}$ is unitary, and

$$\begin{aligned}
\langle u_k|u_k\rangle &= 1 - \sum_{n>N} |u_{nk}|^2 \\
&> 1 - \frac{1}{N} \sum_{n>N} n|u_{nk}|^2 \\
&\geq 1 - \frac{E_{\hat{U}}(M)}{N},
\end{aligned} \tag{8}$$

where we used the definition of $E_{\hat{U}}(M)$ from Eq. (5) in the last line. Moreover, for all $0 \leq i \neq j \leq M$,

$$
\begin{aligned}
|\langle u_i|u_j\rangle| &= \left|\sum_{n=0}^{N} u_{ni}^* u_{nj}\right| \\
&= \left|\sum_{n>N} u_{ni}^* u_{nj}\right| \\
&\leq \sqrt{\sum_{n>N} |u_{ni}|^2 \sum_{n>N} |u_{nj}|^2} \\
&\leq \frac{1}{N}\sqrt{\sum_{n>N} n|u_{ni}|^2 \sum_{n>N} n|u_{nj}|^2} \\
&\leq \frac{E_{\hat{U}}(M)}{N},
\end{aligned}
\tag{9}
$$

where we used the fact that $\hat{U}$ is unitary in the second line, Cauchy–Schwarz inequality in the third line, and the definition of $E_{\hat{U}}(M)$ from Eq. (5) in the last line.

Eqs. (7), (8) and (9) imply that taking N large enough ensures that the $M+1$ first column vectors $(|u_0\rangle, \ldots, |u_M\rangle)$ of the Fock basis matrix of $\Pi_N \hat{U} \Pi_N$ are close to being an orthonormal family. In particular, we define

$$
\delta := \frac{E_{\hat{U}}(M)}{N}, \tag{10}
$$

and assume in what follows that N is chosen large enough so that $\delta < \frac{1}{2M}$. Then,

$$
|\langle u_i|u_j\rangle| \leq \delta \qquad 0 \leq i \neq j \leq M \tag{11}
$$

$$
1-\delta \leq \langle u_k|u_k\rangle \leq 1 \qquad k = 0, \ldots, M. \tag{12}
$$

We also rely on the following technical result, which says that each $|u_j\rangle$ is almost orthogonal to the subspace spanned by $(|u_0\rangle, \ldots, |u_{i-1}\rangle)$, for any $i \leq j$:

Lemma 1. *With $\delta > 0$ defined in Eq. (10) and $\delta < \frac{1}{2M}$, let P_k denotes the orthogonal projector onto* $\mathrm{span}(|u_0\rangle, \ldots, |u_{k-1}\rangle)$ *for all $k = 1, \ldots, M$. For all $0 \leq i \leq j \leq M$, $\langle u_j|P_i|u_j\rangle \leq \delta$. As a result, $\Pi_N \hat{U} \Pi_M$ is column full rank.*

Proof. For all $1 \leq i \leq j \leq M$ we have $\langle u_j|P_i|u_j\rangle \leq \langle u_j|P_j|u_j\rangle$, so it is sufficient to prove the result when $i = j$. For $j = 1, \ldots, M$, defining the normalised projection

$$
|\phi_j\rangle := \frac{P_j|u_j\rangle}{\|P_j|u_j\rangle\|}, \tag{13}
$$

we have $\langle u_j|P_j|u_j\rangle = |\langle u_j|\phi_j\rangle|^2$. Moreover, $|\phi_j\rangle \in \mathrm{span}(|u_0\rangle, \ldots, |u_{j-1}\rangle)$ so we may write $|\phi_j\rangle := \sum_{i=1}^{j-1} \varphi_i |u_i\rangle$, and

we have

$$
\begin{aligned}
1 &= \langle \phi_j | \phi_j \rangle \\
&= \sum_{k,l=0}^{j-1} \varphi_k^* \varphi_l \langle u_k | u_l \rangle \\
&= \sum_{i=0}^{j-1} |\varphi_i|^2 \langle u_i | u_i \rangle + 2\Re \left(\sum_{0 \le k < l \le j-1} \varphi_k^* \varphi_l \langle u_k | u_l \rangle \right) \\
&\geq (1-\delta) \sum_{i=0}^{j-1} |\varphi_i|^2 - 2 \sum_{0 \le k < l \le j-1} |\varphi_k||\varphi_l| |\langle u_k | u_l \rangle| \\
&\geq \sum_{i=0}^{j-1} |\varphi_i|^2 - \delta \sum_{i=0}^{j-1} |\varphi_i|^2 - 2\delta \sum_{0 \le k < l \le j-1} |\varphi_k||\varphi_l| \\
&= \sum_{i=0}^{j-1} |\varphi_i|^2 - \delta \left(\sum_{i=0}^{j-1} |\varphi_i| \right)^2 \\
&\geq (1 - j\delta) \sum_{i=0}^{j-1} |\varphi_i|^2,
\end{aligned}
\tag{14}
$$

where we used Eq. (12) in the third line, Eq. (11) in the fourth line, and Cauchy–Schwarz inequality in the last line. This shows that

$$
\sum_{i=0}^{j-1} |\varphi_i|^2 \le \frac{1}{1 - j\delta},
\tag{15}
$$

and we thus obtain

$$
\begin{aligned}
\langle u_j | P_j | u_j \rangle &= \left| \sum_{i=0}^{j-1} \varphi_i \langle u_j | u_i \rangle \right|^2 \\
&\le \sum_{i=0}^{j-1} |\varphi_i|^2 \sum_{i=0}^{j-1} |\langle u_j | u_i \rangle|^2 \\
&\le \frac{j\delta^2}{1 - j\delta} \\
&\le \delta,
\end{aligned}
\tag{16}
$$

where we used Eqs. (11) and (15) in the third line and $j \le M$ and $\delta < \frac{1}{2M}$ in the last line. This completes the first part of the lemma.

For all $j = 1, \ldots, M$, we obtain $\langle u_j | I - P_j | u_j \rangle = \langle u_j | u_j \rangle - \langle u_j | P_j | u_j \rangle \geq 1 - 2\delta$, so $|u_j\rangle \notin \mathrm{span}(|u_0\rangle, \ldots, |u_{j-1}\rangle)$ and $\Pi_N \hat{U} \Pi_M$ is column full rank. □

Now that these properties are established, recall that we are looking for a unitary operator $\hat{V}_N$ over $\mathcal{H}_N$ such that $\| \Pi_N \hat{U} |\phi\rangle\langle\phi| \hat{U}^\dagger \Pi_N - \hat{V}_N |\phi\rangle\langle\phi| \hat{V}_N^\dagger \|_1$ is uniformly bounded over $\mathcal{H}_M$. To this end, we relate $\Pi_N \hat{U} \Pi_N$ to a unitary operator through the Gram–Schmidt process. Note that we can assume $\Pi_N \hat{U} \Pi_N$ to be full rank without loss of generality, because by Lemma 1, $\Pi_N \hat{U} \Pi_M$ is column full rank and can be completed to a full rank matrix on $\mathcal{H}_N$ which matches with $\Pi_N \hat{U} \Pi_N$ on $\mathcal{H}_N$.

We thus assume $\Pi_N \hat{U} \Pi_N$ to be full rank and write its QR decomposition $\Pi_N \hat{U} \Pi_N = \hat{Q}_N \hat{R}_N$ where $\hat{Q}_N$ is unitary over $\mathcal{H}_N$ and where $\hat{R}_N$ has an upper-triangular matrix in Fock basis given by:

$$
\begin{pmatrix}
\langle e_0 | u_0 \rangle & \langle e_0 | u_1 \rangle & \langle e_0 | u_2 \rangle & \cdots & \langle e_0 | u_M \rangle \\
0 & \langle e_1 | u_1 \rangle & \langle e_1 | u_2 \rangle & \cdots & \langle e_1 | u_N \rangle \\
0 & 0 & \langle e_2 | u_2 \rangle & \cdots & \langle e_2 | u_N \rangle \\
\vdots & \vdots & \vdots & \ddots & \vdots \\
0 & 0 & 0 & \cdots & \langle e_N | u_N \rangle
\end{pmatrix},
\tag{17}
$$

where $(|u_0\rangle, \dots, |u_N\rangle)$ are the column vectors of the Fock basis matrix of $\Pi_N \hat{U} \Pi_N$, and where $(|e_0\rangle, \dots, |e_N\rangle)$ are their orthonormalised version through the Gram–Schmidt process, forming the column vectors of the Fock basis matrix of $\hat{Q}_N$. Recall from Lemma 1 that for all $k = 1, \dots, N$, P_k denotes the orthogonal projector onto $\mathrm{span}(|u_0\rangle, \dots, |u_{k-1}\rangle)$. Let

$$\begin{aligned} |v_0\rangle &:= |u_0\rangle \\ |v_k\rangle &:= (I - P_k)\,|u_k\rangle \,. \end{aligned} \tag{18}$$

The orthonormal vectors $(|e_0\rangle, \dots, |e_N\rangle)$ are then given by

$$|e_k\rangle := \frac{|v_k\rangle}{\|v_k\|}, \tag{19}$$

for all $k = 0, \dots, N$.

We now set $\hat{V}_N = \hat{Q}_N$. For all $|\phi\rangle \in \mathcal{H}_M$, we have

$$\begin{aligned} \|\Pi_N \hat{U}\,|\phi\rangle\langle\phi|\,\hat{U}^\dagger \Pi_N - \hat{V}_N\,|\phi\rangle\langle\phi|\,\hat{V}_N^\dagger\|_1 &= \|\Pi_N \hat{U} \Pi_N\,|\phi\rangle\langle\phi|\,\Pi_N \hat{U}^\dagger \Pi_N - \hat{V}_N\,|\phi\rangle\langle\phi|\,\hat{V}_N^\dagger\|_1 \\ &= \|\hat{Q}_N \hat{R}_N\,|\phi\rangle\langle\phi|\,\hat{R}_N^\dagger \hat{Q}_N^\dagger - \hat{Q}_N\,|\phi\rangle\langle\phi|\,\hat{Q}_N^\dagger\|_1 \\ &= \|\hat{R}_N\,|\phi\rangle\langle\phi|\,\hat{R}_N^\dagger - |\phi\rangle\langle\phi|\,\|_1 \\ &= \|\Pi_M \hat{R}_N \Pi_M\,|\phi\rangle\langle\phi|\,\Pi_M \hat{R}_N^\dagger \Pi_M - |\phi\rangle\langle\phi|\,\|_1, \end{aligned} \tag{20}$$

where we used the fact that the trace distance is unitarily invariant in the third line and the fact that the Fock basis matrix of $\hat{R}_N$ is upper-triangular in the last line.

To conclude, we show that this quantity can be made arbitrarily small uniformly over $\mathcal{H}_M$ for all M, by picking N large enough. To do so, we bound the coefficients of the Fock basis matrix of $\Pi_M \hat{R}_N \Pi_M$. From Eqs. (18) and (19) we have

$$\langle e_i|u_j\rangle = \frac{\langle v_i|u_j\rangle}{\sqrt{\langle v_i|v_i\rangle}}. \tag{21}$$

for all $0 \le i, j \le M$. In particular, for all $0 \le i < j \le M$, with the convention $P_0 = 0$,

$$\begin{aligned} |\langle v_i|u_j\rangle| &= |\langle u_i|I - P_i|u_j\rangle| \\ &\le |\langle u_i|u_j\rangle| + |\langle u_i|P_i|u_j\rangle| \\ &\le \delta + \sqrt{\langle u_i|P_i|u_i\rangle\,\langle u_j|P_i|u_j\rangle} \\ &\le 2\delta, \end{aligned} \tag{22}$$

where we used the triangle inequality in the second line, Eq. (11) in the third line, Cauchy–Schwarz inequality in the fourth line and Lemma 1 twice in the last line. With $\sqrt{\langle v_i|v_i\rangle} = \sqrt{\langle u_i|u_i\rangle - \langle u_i|P_i|u_i\rangle} \ge \sqrt{1 - 2\delta}$ by Eq. (12) and Lemma 1, this yields

$$\begin{aligned} |\langle e_i|u_j\rangle| &= \frac{|\langle v_i|u_j\rangle|}{\sqrt{\langle v_i|v_i\rangle}} \\ &\le \frac{2\delta}{\sqrt{1 - 2\delta}} \\ &\le 4\delta, \end{aligned} \tag{23}$$

where we used $\delta \le \frac{3}{8}$ in the last line. Similarly, for all $k = 0, \dots, M$,

$$\begin{aligned} \langle e_k|u_k\rangle &= \frac{\langle v_k|u_k\rangle}{\sqrt{\langle v_k|v_k\rangle}} \\ &= \frac{\langle u_k|I - P_k|u_k\rangle}{\sqrt{\langle u_k|I - P_k|u_k\rangle}} \\ &= \sqrt{\langle u_k|I - P_k|u_k\rangle} \\ &= \sqrt{\langle u_k|u_k\rangle - \langle u_k|P_k|u_k\rangle}. \end{aligned} \tag{24}$$

Hence, $0 \leq \langle e_k|u_k\rangle \leq 1$ by Eq. (12), and $\langle e_k|u_k\rangle \geq \sqrt{1-2\delta}$ by Eq. (12) and Lemma 1, leading to

$$\begin{aligned} |\langle e_k|u_k\rangle - 1| &\leq 1-\sqrt{1-2\delta} \\ &\leq 2\delta, \end{aligned} \tag{25}$$

where we used $\delta \leq \frac{1}{2}$ in the last line.

To convert these bounds to a bound on the trace distance in Eq. (20), we make use of the following technical result:

Lemma 2 (Trace distance between unnormalised pure states)**.** *Let $\hat{A}$ be a linear operator over $\mathcal{H}_M$ and let $|\phi\rangle \in \mathcal{H}_M$ be a normalised pure state. Then*

$$\|\hat{A}\,|\phi\rangle\langle\phi|\,\hat{A}^\dagger - |\phi\rangle\langle\phi|\,\|_1 = \sqrt{(1+\langle\phi|\hat{A}^\dagger\hat{A}|\phi\rangle)^2 - 4|\,\langle\phi|\hat{A}|\phi\rangle\,|^2}. \tag{26}$$

Proof. We write $\hat{A}\,|\phi\rangle$ in a basis $(|\phi\rangle\,, |\phi^\perp\rangle)$, where $|\phi^\perp\rangle$ is orthogonal to $|\phi\rangle$. The density matrix of $\hat{A}\,|\phi\rangle\langle\phi|\,\hat{A}^\dagger - |\phi\rangle\langle\phi|$ in that basis is given by

$$\begin{pmatrix} |\,\langle\phi|\hat{A}|\phi\rangle\,|^2 - 1 & |\,\langle\phi|\hat{A}|\phi\rangle\,|\sqrt{\langle\phi|\hat{A}^\dagger\hat{A}|\phi\rangle - |\,\langle\phi|\hat{A}|\phi\rangle\,|^2} \\ |\,\langle\phi|\hat{A}|\phi\rangle\,|\sqrt{\langle\phi|\hat{A}^\dagger\hat{A}|\phi\rangle - |\,\langle\phi|\hat{A}|\phi\rangle\,|^2} & \langle\phi|\hat{A}^\dagger\hat{A}|\phi\rangle - |\,\langle\phi|\hat{A}|\phi\rangle\,|^2 \end{pmatrix}, \tag{27}$$

with eigenvalues

$$\lambda_\pm = \frac{1}{2}(\langle\phi|\hat{A}^\dagger\hat{A}|\phi\rangle - 1) \pm \sqrt{\frac{1}{4}(\langle\phi|\hat{A}^\dagger\hat{A}|\phi\rangle - 1)^2 + \langle\phi|\hat{A}^\dagger\hat{A}|\phi\rangle - |\,\langle\phi|\hat{A}|\phi\rangle\,|^2}. \tag{28}$$

Hence,

$$\begin{aligned} \|\hat{A}\,|\phi\rangle\langle\phi|\,\hat{A}^\dagger - |\phi\rangle\langle\phi|\,\|_1 &= |\lambda_+| + |\lambda_-| \\ &= 2\sqrt{\frac{1}{4}(\langle\phi|\hat{A}^\dagger\hat{A}|\phi\rangle - 1)^2 + \langle\phi|\hat{A}^\dagger\hat{A}|\phi\rangle - |\,\langle\phi|\hat{A}|\phi\rangle\,|^2} \\ &= \sqrt{(1+\langle\phi|\hat{A}^\dagger\hat{A}|\phi\rangle)^2 - 4|\,\langle\phi|\hat{A}|\phi\rangle\,|^2}. \end{aligned} \tag{29}$$

□

We apply this lemma to $\hat{A} = \Pi_M \hat{R}_N \Pi_M$. By Eqs. (23,25),

$$|\,\langle i|\hat{A}|j\rangle\,| \leq 4\delta \qquad 0 \leq i \neq j \leq M \tag{30}$$

$$1-2\delta \leq \langle k|\hat{A}|k\rangle \leq 1 \qquad k = 0,\ldots,M. \tag{31}$$

As a result, for $0 \leq i < j \leq N$,

$$\begin{aligned} |\,\langle i|\hat{A}^\dagger\hat{A}|j\rangle\,| &= \left|\sum_{k=0}^{M} \langle i|\hat{A}^\dagger|k\rangle\,\langle k|\hat{A}|j\rangle\right| \\ &\leq \sum_{k=0}^{i-1} |\,\langle i|\hat{A}^\dagger|k\rangle\,||\,\langle k|\hat{A}|j\rangle\,| + |\,\langle i|\hat{A}^\dagger|i\rangle\,||\,\langle i|\hat{A}|j\rangle\,| \\ &\leq 16N\delta^2 + 4\delta \\ &\leq 12\delta, \end{aligned} \tag{32}$$

where we used $\delta < \frac{1}{2M}$ in the last line. Similarly, for all $k = 0,\ldots,M$,

$$\begin{aligned} |\,\langle k|\hat{A}^\dagger\hat{A}|k\rangle\,| &\leq \sum_{l=0}^{k-1} |\,\langle k|\hat{A}^\dagger|l\rangle\,||\,\langle l|\hat{A}|k\rangle\,| + |\,\langle k|\hat{A}^\dagger|k\rangle\,|^2 \\ &\leq 16N\delta^2 + 1 \\ &\leq 1 + 8\delta, \end{aligned} \tag{33}$$

where we used $\delta < \frac{1}{2M}$ in the last line. For $|\phi\rangle := \sum_{k=0}^{M} \phi_k |k\rangle \in \mathcal{H}_M$, we thus have

$$\begin{aligned} |\langle\phi|\hat{A}^\dagger\hat{A}|\phi\rangle| &\leq \sum_{k=0}^{M} |\phi_k|^2 |\langle k|\hat{A}^\dagger\hat{A}|k\rangle| + \sum_{i\neq j} |\phi_i||\phi_j||\langle i|\hat{A}^\dagger\hat{A}|j\rangle| \\ &\leq 1 + 8\delta + 12\delta \sum_{i\neq j} |\phi_i||\phi_j| \\ &\leq 1 + (8+12N)\delta, \end{aligned} \tag{34}$$

where we used Cauchy–Schwarz inequality in the last line. We also have

$$\begin{aligned} |\langle\phi|\hat{A}|\phi\rangle| &= \left|\sum_{k=0}^{M} |\phi_k|^2 \langle k|\hat{A}|k\rangle + \sum_{i\neq j} \phi_i^*\phi_j \langle i|\hat{A}|j\rangle\right| \\ &\geq \sum_{k=0}^{M} |\phi_k|^2 \langle k|\hat{A}|k\rangle - \sum_{i\neq j} |\phi_i||\phi_j||\langle i|\hat{A}|j\rangle| \\ &\geq 1 - 2\delta - 4\delta \sum_{i\neq j} |\phi_i||\phi_j| \\ &\geq 1 - (2+4N)\delta, \end{aligned} \tag{35}$$

where we used Cauchy–Schwarz inequality in the last line. With Lemma 2, this implies

$$\begin{aligned} \|\hat{A}|\phi\rangle\langle\phi|\hat{A}^\dagger - |\phi\rangle\langle\phi|\|_1 &= \sqrt{(1+\langle\phi|\hat{A}^\dagger\hat{A}|\phi\rangle)^2 - 4|\langle\phi|\hat{A}|\phi\rangle|^2} \\ &\leq \sqrt{(2+(8+12M)\delta)^2 - 4(1-(2+4M)\delta)^2} \\ &= \sqrt{\delta}\sqrt{12(4+5M) + 16(3+8M+6M^2)\delta} \\ &\leq \sqrt{\delta}\sqrt{12(12+9M)}, \end{aligned} \tag{36}$$

where we used $\delta < \frac{1}{2M}$ in the last line. Hence, with Eq. (20) we finally obtain

$$\|\Pi_N\hat{U}|\phi\rangle\langle\phi|\hat{U}^\dagger\Pi_N - \hat{V}_N|\phi\rangle\langle\phi|\hat{V}_N^\dagger\|_1 \leq \sqrt{\delta}\sqrt{12(12+9M)}. \tag{37}$$

(iv) Combining Eqs. (4), (6) and (37) yields

$$\begin{aligned} \|(\mathcal{U}-\mathcal{V})(\psi)\|_1 &\leq 4\sqrt{\frac{E}{M}} + 2\sqrt{\frac{E_{\hat{U}}(M)}{N}} + \sqrt{\delta}\sqrt{12(12+9M)} \\ &= 4\sqrt{\frac{E}{M}} + \sqrt{\frac{E_{\hat{U}}(M)}{N}}(2+\sqrt{12(12+9M)}). \end{aligned} \tag{38}$$

where we have used $\delta = \frac{E_{\hat{U}}(M)}{N}$ and where $\mathcal{V} = \hat{V}\cdot\hat{V}^\dagger$ with $\hat{V} = \hat{V}_N \oplus \hat{V}'$, where $\hat{V}_N$ is a unitary operator over $\mathcal{H}_N$ obtained via the orthonormalisation of a column full rank completion of $\Pi_N\hat{U}\Pi_M$ and $\hat{V}'$ is any unitary operator over $\mathrm{Range}(I-\Pi_N)$. Finally, choosing

$$\begin{aligned} M &= \frac{64E}{\epsilon^2} \\ N &= \frac{4E_{\hat{U}}(M)(2+\sqrt{12(12+9M)})^2}{\epsilon^2} = \mathcal{O}\left(\frac{E}{\epsilon^4}E_{\hat{U}}\left(\frac{64E}{\epsilon^2}\right)\right), \end{aligned} \tag{39}$$

ensures $\delta = \frac{E_{\hat{U}}(M)}{N} < \frac{1}{2M}$ and

$$\|(\mathcal{U}-\mathcal{V})(\psi)\|_1 \leq \epsilon. \tag{40}$$

The time complexity of computing $\hat{V}_N$ is given by the time complexity of the QR decomposition [2] of $\Pi_N\hat{U}\Pi_M$, i.e.,

$$\mathcal{O}(NM^2) = \mathcal{O}\left(\frac{NE^2}{\epsilon^4}\right) = \mathcal{O}\left(\frac{E^3}{\epsilon^8}E_{\hat{U}}\left(\frac{64E}{\epsilon^2}\right)\right). \tag{41}$$

□

II. GROWTH OF THE ENERGY IN PHYSICAL UNITARY DYNAMICS

Recall the definition of the maximal amount of energy involved when implementing a physical unitary evolution $\hat{U} \in \mathcal{U}(\mathcal{S})$ of an initial state with a number of particles at most n:

$$E_{\hat{U}}(n) := \sup_{|\psi\rangle \in \mathcal{H}_n} \langle\psi| \hat{U}^\dagger \hat{n} \hat{U} |\psi\rangle < +\infty. \tag{42}$$

We give an explicit construction of physical unitary channels for which this quantity can grow arbitrarily fast with photon number:

1. Pick some sparse countably-infinite subset X of $\mathbb{N}$, e.g., the multiples of 10 excluding zero.
2. Pick some bijection $g : \mathbb{N} \setminus X \to \mathbb{N} \setminus 2^{(X)}$ (which are both countably infinite sets)
3. Now, define a unitary $\hat{U}$ acting on Fock states as

$$\hat{U}|x\rangle = \begin{cases} |2^x\rangle, & \text{if } x \in X \\ |g(x)\rangle, & \text{if } x \in \mathbb{N} \setminus X \end{cases} \tag{43}$$

Here, $\hat{U}$ is physical by construction, because it sends Fock states to Fock states, and $E_{\hat{U}}(n)$ grows at least as fast as 2^n. This reasoning is not specific to the exponential function but can be repeated for any function defined on $\mathbb{N}$, showing that there are physical unitaries $\hat{U}$ for which $E_{\hat{U}}(n)$ grows arbitrarily fast in n.

III. PROOF OF THEOREM 2: FINITE-DIMENSIONAL UNIVERSALITY OF POLYNOMIAL HAMILTONIANS

Hereafter, we write $(\hat{\boldsymbol{q}}, \hat{\boldsymbol{p}}) = (\hat{q}_1, \hat{p}_1, \ldots, \hat{q}_m, \hat{p}_m)$ for $m \geq 1$. We recall Theorem 2 from the main text, extended to the multimode setting:

Theorem 2 (Finite-dimensional universality of polynomial Hamiltonians)**.** *Let $m \geq 1$, let $N_1, \ldots, N_m \in \mathbb{N}$ and let $\hat{H}$ be a Hermitian operator over $\bigotimes_{k=1}^m \mathcal{H}_{N_k}$. There exists a polynomial Hamiltonian $P_{\hat{H}}(\hat{\boldsymbol{q}}, \hat{\boldsymbol{p}})$ of degree at most $3^m N_1 \cdots N_m$ over $\mathcal{H}^{\otimes m}$ such that*

$$P_{\hat{H}}(\hat{\boldsymbol{q}}, \hat{\boldsymbol{p}}) = \hat{H} \oplus \hat{H}', \tag{44}$$

where $\hat{H}'$ is a Hermitian operator over $\mathrm{Range}(I - \bigotimes_{k=1}^m \Pi_{N_k})$*. In particular,*

$$e^{iP_{\hat{H}}(\hat{\boldsymbol{q}}, \hat{\boldsymbol{p}})} |\boldsymbol{\psi}\rangle = e^{i\hat{H}} |\boldsymbol{\psi}\rangle, \tag{45}$$

for all $|\boldsymbol{\psi}\rangle \in \bigotimes_{k=1}^m \mathcal{H}_{N_k}$. Moreover, the polynomial $P_{\hat{H}}$ can be computed efficiently in the size of $\hat{H}$.

Proof sketch. The proof proceeds by showing that any finite-dimensional linear operator can be written as the restriction of an infinite-dimensional operator that is a polynomial in canonical bosonic operators, with the property that it does not mix the finite-dimensional subspace with the rest of the Hilbert space. In other terms, in the single-mode case, if $\hat{A}$ is a linear operator acting on the finite-dimensional Hilbert space $\mathcal{H}_N$, we construct a polynomial $P_{\hat{A}}$ such that

$$P_{\hat{A}}(\hat{q}, \hat{p}) = \Pi_N \hat{A} \Pi_N \oplus (I - \Pi_N) P_{\hat{A}}(\hat{q}, \hat{p})(I - \Pi_N). \tag{46}$$

This construction is based on adding together $(N+1) \times (N+1)$ interpolation polynomials, each reproducing a single entry of the linear operator $\hat{A}$, taking advantage of the sparsity of the canonical operators in Fock basis. Using this result for $\hat{A}$ Hermitian and taking the operator exponential completes the proof in the single-mode case.

The proof then extends to the multimode setting, by taking tensor products and linear combinations of the single-mode polynomials constructed above.

Proof of Theorem 2. We first prove the result in the single-mode setting. Let $N \geq 0$ and let $\hat{A}$ be a linear operator on $\mathcal{H}_N$. We construct a polynomial $P_{\hat{A}}$ such that $P_{\hat{A}}(\hat{q},\hat{p}) = \Pi_N \hat{A} \Pi_N + (I - \Pi_N) P_{\hat{A}}(\hat{q},\hat{p})(I - \Pi_N)$. To do so, we only need to treat the cases where $\hat{A} = |n\rangle\langle n|$, $\hat{A} = |n\rangle\langle n+k|$, and $\hat{A} = |n+k\rangle\langle n|$, for $n = 0,\dots,N$, $k = 1,\dots,N$ and $n+k \leq N$, since the general case is obtained by taking linear combinations of these elementary cases.

For the case $\hat{A} = |n\rangle\langle n|$, for $n = 0,\dots,N$, we pick $P_{|n\rangle\langle n|}(\hat{q},\hat{p}) = P_n(\hat{n})$, where $\hat{n} = \hat{a}^\dagger\hat{a} = \frac{1}{2}(\hat{q}^2 + \hat{p}^2 - \hat{I})$ and where P_n is a polynomial satisfying $P_n(m) = \delta_{nm}$ for all integers $0 \leq m \leq N$. Using the Lagrange interpolation polynomial for these values, we thus define:

$$P_n(X) := \prod_{\substack{k=0\\k\neq n}}^{N} \frac{X-k}{n-k}. \tag{47}$$

Then, for all $m \in \mathbb{N}$,

$$\begin{aligned} P_{|n\rangle\langle n|}(\hat{q},\hat{p})\,|m\rangle &= P_n(\hat{n})\,|m\rangle \\ &= \left(\prod_{\substack{k=0\\k\neq n}}^{N} \frac{\hat{n}-k}{n-k}\right)|m\rangle \\ &= \left(\prod_{\substack{k=0\\k\neq n}}^{N} \frac{m-k}{n-k}\right)|m\rangle \\ &= \begin{cases} |n\rangle & m = n \\ 0 & m \neq n \quad \text{and} \quad m \leq N \\ \left(\prod_{\substack{k=0\\k\neq n}}^{N} \frac{m-k}{n-k}\right)|m\rangle & m > N. \end{cases} \end{aligned} \tag{48}$$

By construction, $P_{|n\rangle\langle n|}(\hat{q},\hat{p}) = \Pi_N\,|n\rangle\langle n|\,\Pi_N + (I - \Pi_N)P_{|n\rangle\langle n|}(\hat{q},\hat{p})(I - \Pi_N)$, and $P_{|n\rangle\langle n|}$ has degree $2N$.

For the case $\hat{A} = |n\rangle\langle n+k|$, for $n = 0,\dots,N$, $k = 1,\dots,N$ and $n+k \leq N$, we pick $P_{|n\rangle\langle n+k|}(\hat{q},\hat{p}) = P_{n,k}(\hat{n})\hat{a}^k$, where $P_{n,k}$ is a polynomial to be determined. For all $i,j \in \mathbb{N}$,

$$\begin{aligned} \langle i|P_{|n\rangle\langle n+k|}(\hat{q},\hat{p})|j\rangle &= \langle i|P_{n,k}(\hat{n})\hat{a}^k|j\rangle \\ &= \begin{cases} P_{n,k}(i)\sqrt{\frac{(i+k)!}{i!}} & j = i+k \\ 0 & \text{otherwise.} \end{cases} \end{aligned} \tag{49}$$

Hence, to ensure that $P_{|n\rangle\langle n+k|}(\hat{q},\hat{p}) = \Pi_N\,|n\rangle\langle n+k|\,\Pi_N \oplus (I - \Pi_N)P_{|n\rangle\langle n+k|}(\hat{q},\hat{p})(I - \Pi_N)$, we choose

$$P_{n,k}(i) = \begin{cases} \sqrt{\frac{n!}{(n+k)!}} & i = n \\ 0 & i \neq n \quad \text{and} \quad i \leq N. \end{cases} \tag{50}$$

Note that enforcing the last condition for $i \leq N-k$ would be sufficient to strictly reproduce the action of the target operator on the finite-dimensional subspace $\mathrm{Range}(\Pi_N)$, but enforcing this condition for $i \leq N$ instead ensures that the corresponding infinite-dimensional operator also has the desired block-diagonal structure in Fock basis. Using the Lagrange interpolation polynomial for these values, we thus define:

$$\begin{aligned} P_{n,k}(X) :=& \sqrt{\frac{n!}{(n+k)!}} \prod_{\substack{m=0\\m\neq n}}^{N} \frac{X-m}{n-m} \\ =& \sqrt{\frac{n!}{(n+k)!}} P_n(X). \end{aligned} \tag{51}$$

This polynomial has degree N, so $P_{|n\rangle\langle n+k|}(\hat{q},\hat{p}) = P_{n,k}(\hat{n})\hat{a}^k$ has degree $2N + k \leq 3N$.

For the case $\hat{A} = |n+k\rangle\langle n|$, for $n = 0, \ldots, N$, $k = 1, \ldots, N$ and $n+k \leq N$, we pick $P_{|n+k\rangle\langle n|}(\hat{q}, \hat{p}) = P_{|n\rangle\langle n+k|}(\hat{q}, \hat{p})^\dagger$, which also has degree $2N + k \leq 3N$.

For the general case, we write $\hat{A} = \sum_{n=0}^N a_{nn} |n\rangle\langle n| + \sum_{0\leq i<j\leq N} a_{ij} |i\rangle\langle j| + a_{ji} |j\rangle\langle i|$. Combining the previous cases, we obtain

$$P_{|i\rangle\langle j|}(\hat{q}, \hat{p}) = \sqrt{\frac{\min(i,j)!}{\max(i,j)!}}\hat{a}^{\dagger(\max(i,j)-j)} P_{\min(i,j)}(\hat{n})\hat{a}^{\max(i,j)-i}, \tag{52}$$

where P_n is defined in Eq. (47), and we define

$$\begin{aligned} P_{\hat{A}}(\hat{q}, \hat{p}) &:= \sum_{0\leq i,j\leq N} a_{ij} P_{|i\rangle\langle j|}(\hat{q}, \hat{p}) \\ &= \sum_{n=0}^N a_{nn} P_n(\hat{n}) + \sum_{0\leq i<j\leq N} a_{ij}\sqrt{\frac{i!}{j!}} P_i(\hat{n})\hat{a}^{j-i} + a_{ji}\sqrt{\frac{i!}{j!}}\hat{a}^{\dagger(j-i)} P_i(\hat{n}), \end{aligned} \tag{53}$$

which has degree less or equal to $3N$. By construction, we have

$$P_{\hat{A}}(\hat{q}, \hat{p}) = \Pi_N \hat{A} \Pi_N + (I - \Pi_N) P_{\hat{A}}(\hat{q}, \hat{p})(I - \Pi_N), \tag{54}$$

and $P_{\hat{A}}(\hat{q}, \hat{p})$ is Hermitian when $\hat{A}$ is Hermitian.

Let $\hat{U}$ be a unitary operator over $\mathcal{H}_M$. There exists a Hermitian operator $\hat{H}$ over $\mathcal{H}_M$ such that $\hat{U} = e^{i\hat{H}}$. Then, $P_{\hat{H}}(\hat{q}, \hat{p}) = \Pi_N \hat{H} \Pi_N + (I - \Pi_N) P_{\hat{H}}(\hat{q}, \hat{p})(I - \Pi_N)$ stabilises Range(Π_N) so

$$\begin{aligned} e^{iP_{\hat{H}}(\hat{q}, \hat{p})}\Pi_N &= \Pi_N e^{iP_{\hat{H}}(\hat{q}, \hat{p})}\Pi_N \\ &= e^{i\Pi_N \hat{H} \Pi_N} \\ &= \Pi_N e^{i\hat{H}} \Pi_N \\ &= \hat{U}. \end{aligned} \tag{55}$$

We now turn to the multimode setting. Let $m \geq 1$ be the number of modes, and let $\hat{A}$ be a linear operator over $\bigotimes_{k=1}^m \mathcal{H}_{N_k}$, for $N_1, \ldots, N_m \in \mathbb{N}$. Writing $\hat{A} = \sum_{k=0}^m \sum_{i_k, j_k=0}^{N_k} a_{\boldsymbol{ij}} |\boldsymbol{i}\rangle\langle \boldsymbol{j}|$ and $(\hat{\boldsymbol{q}}, \hat{\boldsymbol{p}}) = (\hat{q}_1, \hat{p}_1, \ldots, \hat{q}_m, \hat{p}_m)$, we define

$$P_{\hat{A}}(\hat{\boldsymbol{q}}, \hat{\boldsymbol{p}}) := \sum_{k=0}^m \sum_{i_k, j_k=0}^{N_k} a_{\boldsymbol{ij}} \bigotimes_{k=1}^m P_{|i_k\rangle\langle j_k|}(\hat{q}_k, \hat{p}_k), \tag{56}$$

which is a polynomial of degree at most $3^m \times N_1 \cdots N_m$, where $P_{|i_k\rangle\langle j_k|}(\hat{q}_k, \hat{p}_k)$ is the single-mode polynomial defined in Eq. (52). By Eq. (54), this polynomial satisfies

$$P_{\hat{A}}(\hat{\boldsymbol{q}}, \hat{\boldsymbol{p}}) = \sum_{k=0}^m \sum_{i_k, j_k=0}^{N_k} a_{\boldsymbol{ij}} \bigotimes_{k=1}^m \left[\Pi_{N_k} |i_k\rangle\langle j_k| \Pi_{N_k} + (I - \Pi_{N_k}) P_{|i_k\rangle\langle j_k|}(\hat{q}_k, \hat{p}_k)(I - \Pi_{N_k})\right]. \tag{57}$$

Expanding the tensor product leads to a single product term of the form

$$\bigotimes_{k=1}^m (\Pi_{N_k} |i_k\rangle\langle j_k| \Pi_{N_k}) = \left(\bigotimes_{k=1}^m \Pi_{N_k}\right) |\boldsymbol{i}\rangle\langle \boldsymbol{j}| \left(\bigotimes_{k=1}^m \Pi_{N_k}\right), \tag{58}$$

while all the other product terms contain at least one term of the form $(I - \Pi_{N_k}) P_{|i_k\rangle\langle j_k|}(\hat{q}_k, \hat{p}_k)(I - \Pi_{N_k})$, for some $k \in \{1, \ldots, m\}$. As a consequence, these terms can be all be expressed as operators over Range$(I - \bigotimes_{k=1}^m \Pi_{N_k})$, and thus

$$\begin{aligned} P_{\hat{A}}(\hat{\boldsymbol{q}}, \hat{\boldsymbol{p}}) &= \sum_{k=0}^m \sum_{i_k, j_k=0}^{N_k} a_{\boldsymbol{ij}} \left(\bigotimes_{k=1}^m \Pi_{N_k}\right) |\boldsymbol{i}\rangle\langle \boldsymbol{j}| \left(\bigotimes_{k=1}^m \Pi_{N_k}\right) + \left(I - \bigotimes_{k=1}^m \Pi_{N_k}\right) P_{\hat{A}}(\hat{\boldsymbol{q}}, \hat{\boldsymbol{p}}) \left(I - \bigotimes_{k=1}^m \Pi_{N_k}\right) \\ &= \left(\bigotimes_{k=1}^m \Pi_{N_k}\right) \sum_{k=0}^m \sum_{i_k, j_k=0}^{N_k} a_{\boldsymbol{ij}} |\boldsymbol{i}\rangle\langle \boldsymbol{j}| \left(\bigotimes_{k=1}^m \Pi_{N_k}\right) + \left(I - \bigotimes_{k=1}^m \Pi_{N_k}\right) P_{\hat{A}}(\hat{\boldsymbol{q}}, \hat{\boldsymbol{p}}) \left(I - \bigotimes_{k=1}^m \Pi_{N_k}\right) \\ &= \left(\bigotimes_{k=1}^m \Pi_{N_k}\right) \hat{A} \left(\bigotimes_{k=1}^m \Pi_{N_k}\right) + \left(I - \bigotimes_{k=1}^m \Pi_{N_k}\right) P_{\hat{A}}(\hat{\boldsymbol{q}}, \hat{\boldsymbol{p}}) \left(I - \bigotimes_{k=1}^m \Pi_{N_k}\right). \end{aligned} \tag{59}$$

Moreover, this operator is Hermitian when $\hat{A}$ is Hermitian. With the same derivation as in Eq. (55) this concludes the proof.

□

IV. PROOF OF THEOREM 4: SOLOVAY–KITAEV THEOREM FOR POLYNOMIAL HAMILTONIANS

We give a formal version of Theorem 4 from the main text:

Theorem 4 (Solovay–Kitaev theorem for polynomial Hamiltonians). *Let $E > 0$, $\epsilon > 0$ and $N \geq \frac{64E}{\epsilon^2} \in \mathbb{N}$. Let $\mathcal{G}$ be a finite set of unitary operators over $\mathcal{H}_N$ generating a dense subset of $\mathcal{U}(\mathcal{H}_N)$ and let $\mathcal{P}$ be its realization with polynomial Hamiltonians from Theorem 2. There is a constant c such that for any physical unitary operator $\hat{U} \in \mathcal{U}(\mathcal{S})$ with (E,ϵ)-approximate effective dimension $N+1$, there exists a finite sequence $\hat{V}$ of gates from $\mathcal{P}$ of length $\mathcal{O}(\log^c(1/\epsilon))$ and such that $\|\mathcal{U} - \mathcal{V}\|_\diamond^E \leq 2\epsilon$, where $\mathcal{U} = \hat{U} \cdot \hat{U}^\dagger$ and $\mathcal{V} = \hat{V} \cdot \hat{V}^\dagger$.*

Proof. The physical unitary operator $\hat{U}$ has (E,ϵ)-*approximate effective dimension* $N+1$, so with Theorem 1 there exists a unitary operator $\hat{V}_N$ over $\mathcal{H}_N$ such that for any unitary operator $\hat{V}'$ over $\mathrm{Range}(I - \Pi_N)$,

$$\sup_{\langle\psi|\hat{n}|\psi\rangle \leq E} D[(\hat{V}_N \oplus \hat{V}')\,|\psi\rangle\,, \hat{U}\,|\psi\rangle] \leq \frac{\epsilon}{2}, \tag{60}$$

where we have expressed the energy-constrained diamond norm using the trace distance $D(\cdot,\cdot) = \frac{1}{2}\|\cdot - \cdot\|_1$ as in Eq. (3).

The Solovay–Kitaev theorem for qudits [3, Theorem 1] of dimension $N+1$ ensures that there is a constant c such that there exists a finite sequence $\hat{S}$ of gates from $\mathcal{G}$ of length $\mathcal{O}(\log^c(1/\epsilon))$ and such that

$$\sup_{|\phi\rangle \in \mathcal{H}_N} D(\hat{V}_N\,|\phi\rangle\,, \hat{S}\,|\phi\rangle) \leq \frac{\epsilon}{4}, \tag{61}$$

and thus

$$\begin{aligned} |\,\langle\phi|\hat{V}_N^\dagger \hat{S}|\phi\rangle\,| &= \sqrt{1 - D(\hat{V}_N\,|\phi\rangle\,, \hat{S}\,|\phi\rangle)} \\ &\geq \sqrt{1 - \frac{\epsilon^2}{16}}, \end{aligned} \tag{62}$$

for all $|\phi\rangle \in \mathcal{H}_N$.

Now for all $|\psi\rangle = \sum_{n\geq 0} \psi_n\,|n\rangle \in \mathcal{H}$ such that $\langle\psi|\hat{n}|\psi\rangle \leq E$,

$$\begin{aligned} \langle\psi|\Pi_N|\psi\rangle &= \sum_{n=0}^{N} |\psi_n|^2 \\ &= 1 - \sum_{n>N} |\psi_n|^2 \\ &\geq 1 - \sum_{n>N} \frac{n}{N}|\psi_n|^2 \\ &\geq 1 - \frac{1}{N}\sum_{n\geq 0} n|\psi_n|^2 \\ &\geq 1 - \frac{E}{N}. \end{aligned} \tag{63}$$

Moreover, for all $|\psi\rangle = \sum_{n\geq 0} \psi_n\,|n\rangle \in \mathcal{H}$ such that $\langle\psi|\hat{n}|\psi\rangle \leq E$, and for any unitary operator $\hat{V}'$ on $\mathrm{Range}(I - \Pi_N)$,

$$\begin{aligned} D[(\hat{V}_N \oplus \hat{V}')\,|\psi\rangle\,, (\hat{S} \oplus \hat{V}')\,|\psi\rangle] &= \sqrt{1 - |\,\langle\psi|(\hat{V}_N \oplus \hat{V}')^\dagger(\hat{S} \oplus \hat{V}')|\psi\rangle\,|^2} \\ &= \sqrt{1 - |\,\langle\psi|(\hat{V}_N^\dagger \hat{S} \oplus \hat{I})|\psi\rangle\,|^2} \\ &= \sqrt{1 - |\,\langle\psi|\Pi_N \hat{V}_N^\dagger \hat{S} \Pi_N|\psi\rangle + \langle\psi|I - \Pi_N|\psi\rangle\,|^2} \\ &= \sqrt{1 - |\,\langle\psi|\Pi_N|\psi\rangle\,\langle\psi_N|\hat{V}_N^\dagger \hat{S}|\psi_N\rangle + 1 - \langle\psi|\Pi_N|\psi\rangle\,|^2}, \end{aligned} \tag{64}$$

where we have defined $|\psi_N\rangle := \Pi_N |\psi\rangle / \|\Pi_N |\psi\rangle\| \in \mathcal{H}_N$. Using the reverse triangle inequality $|a+b| \geq ||a|-|b||$ we obtain

$$
\begin{aligned}
D[(\hat{V}_N \oplus \hat{V}')|\psi\rangle, (\hat{S} \oplus \hat{V}')|\psi\rangle] &\leq \sqrt{1 - [\langle\psi|\Pi_N|\psi\rangle\, |\langle\psi_N|\hat{V}_N^\dagger \hat{S}|\psi_N\rangle| - (1 - \langle\psi|\Pi_N|\psi\rangle)]^2} \\
&\leq \sqrt{1 - \left[\left(1 - \frac{E}{N}\right)\sqrt{1 - \frac{\epsilon^2}{16}} - \frac{E}{N}\right]^2} \\
&= \sqrt{\frac{\epsilon^2}{16} + \frac{2E}{N}\left(\sqrt{1 - \frac{\epsilon^2}{16}} + 1 - \frac{\epsilon^2}{16}\right) - \frac{E^2}{N^2}\left(1 + \sqrt{1 - \frac{\epsilon^2}{16}}\right)^2} \\
&\leq \sqrt{\frac{\epsilon^2}{16} + \frac{4E}{N}} \\
&\leq \frac{\epsilon}{4} + 2\sqrt{\frac{E}{N}} \\
&\leq \frac{\epsilon}{2},
\end{aligned}
\tag{65}
$$

where we have used Eqs. (62) and (63) in the second line and $N \geq \frac{64E}{\epsilon^2}$ in the last line. Combining this with Eq. (60) and the triangle inequality we obtain

$$
\sup_{\langle\psi|\hat{n}|\psi\rangle \leq E} D[(\hat{S} \oplus \hat{V}')|\psi\rangle, \hat{U}|\psi\rangle] \leq \epsilon, \tag{66}
$$

for any unitary operator $\hat{V}'$ over Range$(I - \Pi_N)$.

Let s denote the length of the sequence $\hat{S}$ given by the qudit Solovay–Kitaev theorem. For each unitary gate $\hat{G}_k \in \mathcal{G}$ over $\mathcal{H}_N$ appearing in the sequence $\hat{S} = \hat{G}_s \cdots \hat{G}_1$, there exists a Hermitian operator $\hat{H}_k$ over $\mathcal{H}_N$ such that $\hat{G}_k = e^{i\hat{H}_k}$, which can be computed efficiently in N, for instance by diagonalizing $\hat{G}_k$. For each gate, Theorem 2 provides a realization generated by a polynomial Hamiltonian $P_{\hat{H}_k}(\hat{q},\hat{p})$ of degree $3N$, such that

$$
P_{\hat{H}_k}(\hat{q},\hat{p}) = \hat{H}_k \oplus \hat{H}'_k, \tag{67}
$$

where $\hat{H}'_k$ is a Hermitian operator over Range$(I - \Pi_N)$, and

$$
e^{iP_{\hat{H}_k}(\hat{q},\hat{p})}\Pi_N = \hat{G}_k, \tag{68}
$$

so that

$$
e^{iP_{\hat{H}_s}(\hat{q},\hat{p})} \cdots e^{iP_{\hat{H}_1}(\hat{q},\hat{p})} = \hat{S} \oplus \hat{V}', \tag{69}
$$

where $\hat{V}'$ is a unitary operator over Range$(I - \Pi_N)$. With Eq. (66) we finally obtain

$$
\sup_{\langle\psi|\hat{n}|\psi\rangle \leq E} D[e^{iP_{\hat{H}_s}(\hat{q},\hat{p})} \cdots e^{iP_{\hat{H}_1}(\hat{q},\hat{p})}|\psi\rangle, \hat{U}|\psi\rangle] \leq \epsilon, \tag{70}
$$

which concludes the proof. □

[1] S. Becker, N. Datta, L. Lami, and C. Rouzé, Physical Review Letters **126**, 190504 (2021).
[2] G. H. Gloub and C. F. Van Loan, Johns Hopkins Universtiy Press, 3rd edtion (1996).
[3] C. M. Dawson and M. A. Nielsen, arXiv preprint quant-ph/0505030 (2005), 10.48550/arXiv.quant-ph/0505030.
